# Integrated analysis with iCM-SANS, SAXS and MD simulations for dynamics of multi-domain proteins

**DOI:** 10.1107/S1600576726005832

**Published:** 2026-07-29

**Authors:** Aya Okuda, Rintaro Inoue, Minami Kurokawa, Anne Martel, Lionel Porcar, Rikuto Osaki, Kaori Fukuzawa, Kevin L. Weiss, Sai Venkatesh Pingali, Reiko Urade, Masaaki Sugiyama

**Affiliations:** a Institute for Integrated Radiation and Nuclear Science Kyoto University 2-1010 Asashiro-nishi Kumatori Sennan-gun Osaka590-0494 Japan; b Institut Laue–Langevin 71 avenue de Martyrs Grenoble38042 France; c Graduate School of Pharmaceutical Sciences The University of Osaka 1-6 Yamadaoka Suita Osaka565-0871 Japan; d Neutron Scattering Division Oak Ridge National Laboratory 1 Bethel Valley Road Oak Ridge TN37831 USA; Uppsala University, Sweden; The European Extreme Light Infrastructure, Czechia

**Keywords:** multi-domain proteins, protein ligation, inverse contrast-matching small-angle neutron scattering, iCM-SANS, size exclusion chromatography, SEC-SANS, small-angle X-ray scattering, SAXS, molecular dynamics simulations

## Abstract

Multi-domain proteins (MDPs) adopt diverse conformations arising from cooperative inter-domain motions, and such dynamics are coupled to their biological functions. Quantitative characterization of these motions is crucial for elucidating their functional mechanisms. Although small-angle X-ray scattering (SAXS) provides information on overall domain arrangement, the limited experimental constraints hinder reliable discrimination of conformational ensembles derived from molecular dynamics (MD) simulations. To address this limitation, complementary experimental constraints that offer domain-selective structural information are required. Inverse contrast-matching small-angle neutron scattering (iCM-SANS), combined with segmental deuteration, enables selective observation of individual domains and provides such complementary constraints. However, practical strategies for preparing segmentally deuterated MDPs have yet to be established. Here, we develop an experimental protocol that integrates controlled protein deuteration with high-efficiency multi-step protein ligation to generate a segmentally deuterated MDP. The combined use of SAXS and iCM-SANS yields complementary structural constraints that enhance discrimination of MD-derived conformational ensembles. This protocol expands the applicability of domain-selective observation, enabling analysis of dynamics in MDPs.

## Abstract 2

Segmental deuteration, combined with inverse contrast-matching small-angle neutron scattering (iCM-SANS) and small-angle X-ray scattering (SAXS), provides structural constraints that improve discrimination of conformation ensembles of multi-domain proteins derived from molecular dynamics (MD) simulations.

## 1. Introduction

Multi-domain proteins (MDPs) consist of multiple independently folded structural units (domains) within a single polypeptide chain. When the intervening linkers in MDPs are flexible, domain–domain arrangements can vary widely, leading to diverse conformations. Previous studies have suggested that their functions are not determined solely by the tertiary structures of individual domains but depend strongly on inter-domain arrangements and their cooperative domain dynamics (Inoue *et al.*, 2010 ▸; Olsson & Wolf-Watz, 2010 ▸; Roy *et al.*, 2016 ▸; Vishwanath *et al.*, 2018 ▸; Inoue *et al.*, 2023 ▸; Biehl *et al.*, 2025 ▸). Accordingly, elucidation of MDP functional mechanisms necessitates quantitative characterization of their dynamics under near-physiological solution conditions.

Small-angle X-ray or neutron scattering (SAXS/SANS) techniques provide spatially and temporally averaged information on the molecular shape and structure of the sample in solution (Guinier *et al.*, 1955 ▸; Svergun & Koch, 2003 ▸; Honecker *et al.*, 2022 ▸). As a result of this capability, SAXS has been extensively employed as a powerful tool for structural analyses of biomacromolecules in solution and applied to the structural characterization of MDPs (Neylon, 2008 ▸; Hou *et al.*, 2020 ▸; Kawamukai *et al.*, 2024 ▸). Furthermore, recent advances integrating SAXS with molecular dynamics (MD) simulations have enabled analyses of dynamics for less flexible MDPs, such as simple two-domain proteins (Oroguchi *et al.*, 2009 ▸; de Souza Degenhardt *et al.*, 2021 ▸; Shukla *et al.*, 2025 ▸). In contrast, these approaches still face substantial limitations for more complex MDPs. As the number of domains increases, the internal degrees of freedom arising from domain correlations expand the conformational degrees of freedom, resulting in the emergence of more complex conformational ensembles. Consequently, conformational ensembles derived from independent MD simulations can produce indistinguishable SAXS profiles despite different domain arrangements (Shevchuk & Hub, 2017 ▸). This degeneracy makes it difficult to discriminate appropriate ensembles solely on the basis of SAXS data (Tuukkanen *et al.*, 2017 ▸), hindering accurate characterization of the dynamics of MDPs. Overcoming this limitation requires integration of SAXS with complementary techniques that directly report on inter-domain arrangements. Such information can subsequently be incorporated as additional experimental constraints.

Neutrons exhibit distinct scattering lengths for hydrogen and its isotope deuterium, and this property enables modulation of the difference in scattering length density (SLD) between the solvent and the solute (Heller, 2010 ▸; Krueger, 2022 ▸). Consequently, SANS provides structural information that cannot be obtained from SAXS and has been established as a complementary technique for the structural analysis of biomacromolecules in solution (Svergun *et al.*, 1998 ▸; Sugiyama *et al.*, 2015 ▸; Josts *et al.*, 2018 ▸). Recent advances in protein deuteration techniques, which enable the modulation of the SLD of the solute, have facilitated the preparation of proteins with controlled degrees of deuteration (Okuda *et al.*, 2021*a* ▸). In particular, the SLD of partially deuterated (*ca* 75%) proteins matches that of 100% D_2_O, which has minimal incoherent background scattering. Under this contrast-matching condition, such proteins become ‘scatteringly invisible’ in SANS measurements (Sugiyama *et al.*, 2014 ▸). Inverse contrast-matching small-angle neutron scattering (iCM-SANS) leverages this principle to achieve selective observation of target components in complexes or multicomponent systems with high signal-to-noise ratios. The combined use of SAXS and iCM-SANS data as constraints in structural modeling and/or MD analyses has successfully elucidated both the structure and the dynamics of a protein complex in solution (Yunoki *et al.*, 2022 ▸). Thus, integration of SAXS and iCM-SANS offers a powerful strategy for dynamical analyses of MDPs.

Cooperative dynamics between spatially separated, non-contiguous domains play crucial roles in the functional regulation of MDPs (Yu *et al.*, 2010 ▸; Liu & Thirumalai, 2022 ▸; Agam *et al.*, 2024 ▸). Investigation of such dynamics requires segmentally deuterated MDPs, where the domains of interest are hydrogenated and the remaining domains are partially deuterated. In a pioneering approach, Sonntag *et al.* (2017 ▸) demonstrated that a single-step protein ligation enables selective observation of a contiguous domain using SANS; however, extension of this strategy to spatially separated, non-contiguous domains remains unestablished. Preparation of segmentally deuterated MDPs targeting non-contiguous domains requires robust, high-efficiency, multi-step protein ligation strategies beyond current methods. Combining segmental deuteration of MDPs targeting selective observation of spatially separated, non-contiguous domains with iCM-SANS enables dynamical analysis and provides domain-resolved structural information relevant to functional mechanisms.

In this study, we selected ER-60 (ERp57) (Urade *et al.*, 2004 ▸; Ellgaard & Ruddock, 2005 ▸; Jessop *et al.*, 2007 ▸; Dong *et al.*, 2009 ▸; PDB ID 3f8u) as a model MDP for the following two reasons. Our previous study demonstrated that SAXS-MD analysis alone cannot provide a uniquely determined conformational ensemble for ER-60, which comprises four domains (Shimizu *et al.*, 2022 ▸), necessitating the incorporation of domain-selective structural information as complementary constraints. From a biological perspective, the terminal **a** and **a**′ domains of ER-60 contain catalytically active CGHC motifs, and their cooperative domain motions have been suggested to be involved in the functional regulation of ER-60 (Okuda *et al.*, 2021*b* ▸). Selective observation of these domains is therefore both biologically significant and well suited for validating the present methodology.

By integrating our developed high-efficiency, multi-step protein ligation strategy (Okuda *et al.*, 2023 ▸) with a controlled protein deuteration technique (Okuda *et al.*, 2021*a* ▸), we prepared segmentally deuterated ER-60 that facilitates the analysis of inter-domain dynamics between non-contiguous domains. Through iCM-SANS and SAXS measurements, together with correlation analyses based on MD simulations, we propose a methodological protocol that allows improved discrimination of MD-derived conformational ensembles of MDPs through domain-selective structural constraints.

## 2. Materials and methods

### 2.1. Establishment of expression systems for ER-60 ligation-mutant

To selectively observe the terminal **a** and **a**′ domains by iCM-SANS, ER-60 was decomposed into three fragments, the **a**, **bb**′ and **a**′ domains, which were subsequently reconstituted into full-length ER-60 through enzymatic protein ligation. In enzymatic protein ligation, an amino acid sequence recognized by the ligation enzyme is typically retained in the final ligation product. In the present system, an asparagine–glycine–leucine (NGL) sequence remains at the ligation junction. It is therefore essential to verify that this ligation-derived sequence does not perturb the native structure of the target protein. To assess the structural impact of the NGL sequence on full-length ER-60, a corresponding ER-60 variant containing the same substitutions (hereafter referred to as ER-60 ligation-mutant; **a**-**bb**′-**a**′) was generated in advance. Specifically, four amino acid substitutions (D122N, I124L, R363N and Y364G) were introduced to create the NGL sequence within the ER-60 backbone.

The DNA plasmid encoding ER-60 ligation-mutant (**a**-**bb**′-**a**′) was generated from the pET-20*b*(+) plasmid harboring full-length wild-type human ER-60 (Urade *et al.*, 1997 ▸) by site-directed mutagenesis polymerase chain reaction (PCR) using oligonucleotide primer pairs (D122N/I124L: 5′-GCTAATGGACTGGTCAGCCACTTGAAGAAG-3′ and 5′-GACCAGTCCATTAGCAGTCCTAGGTCCATC-3′; R363N/Y364G: 5′-GAAGAACGGCCTGAAGTCTGAACCTATC-3′ and 5′-TTCAGGCCGTTCTTCAGATTGCCATCAAAG-3′) and PrimeSTAR Max DNA Polymerase (TaKaRa Bio Inc., Japan). The resulting PCR products were directly transformed into *Escherichia coli* TOP10F, and circular expression plasmids were obtained. The expression plasmids were transformed into *E. coli* BL21 (DE3) strain (Novagen, Germany).

### 2.2. Preparation of recombinant hydrogenated and deuterated proteins

In iCM-SANS, hydrogenated proteins are scatteringly visible whereas partially deuterated proteins are scatteringly invisible in 100% D_2_O buffer (see Section 3.1 for details). Therefore, the **a** and **a**′ domains were prepared as hydrogenated proteins, whereas the **bb**′ domains were prepared as partially deuterated proteins. The recombinant proteins were expressed using an *E. coli* expression system. Detailed construct information is provided by Okuda *et al.* (2023 ▸). The expression of hydrogenated recombinant wild-type ER-60, ligation-mutant, and **a** and **a**′ domain fragments was induced in H_2_O Luria–Bertani (LB) culture containing 0.1 m*M* iso­propyl-β-d-thio­galactoside and corresponding antibiotics (wild-type ER-60 and ligation-mutant: 100 µg mL^−1^ ampicillin; domain fragments: 15 µg mL^−1^ kanamycin) at 16 °C for 64 h. *E. coli* cells were collected by centrifugation at 6500*g* for 20 min at 4 °C and disrupted by sonication in 20 m*M* HEPES buffer (pH 6.8) containing 50 m*M* KCl, 5 m*M* EDTA and 1 m*M* phenyl­methyl­sulfonyl fluoride. The homogenate was centrifuged at 26000*g* for 20 min at 4 °C. Soluble proteins in the supernatants were applied to a packed column with TOYOPEARL AF-Heparin 650M (Tosoh Corporation, Japan). The column was washed with 20 m*M* HEPES buffer (pH 6.8) containing 50 m*M* KCl and 5 m*M* EDTA. Proteins bound to the resin were eluted with 20 m*M* HEPES buffer (pH 6.8) containing 400 m*M* KCl and 5 m*M* EDTA. The eluted proteins were subsequently purified by ion-exchange column chromatography on a Resource Q column (GE Healthcare) with 20 m*M* Tris–HCl buffer (pH 8.0), followed by gel filtration chromatography on a Superdex 200 Increase 10/300 GL column (GE Healthcare) with 20 m*M* Tris–HCl buffer (pH 8.0) containing 150 m*M* NaCl. In the C-side **a**′ domains, the His_6_-Smt3 tag was cleaved by treatment with SUMO protease (LifeSensors, USA) at 4 °C overnight. The cleaved tag was removed from the samples by applying them to His60 Ni Superflow Resin (TaKaRa BIO Inc.) and then performing size exclusion chromatography (SEC) on a Superdex 200 Increase 10/300 GL column with 20 m*M* Tris–HCl buffer (pH 8.0) containing 150 m*M* NaCl and 1 m*M* CaCl_2_. The partially deuterated recombinant **bb**′ domain fragment of ER-60 was expressed as previously described (Okuda *et al.*, 2021*a* ▸). *E. coli* cells were cultured in 30% D_2_O LB culture solution for 12 h at 37 °C. Then, 100 µL of the 30% D_2_O LB culture solution of *E. coli* was added to 5 mL of 60% D_2_O LB culture solution and cultured for 12 h at 37 °C. The cells in 60% D_2_O LB culture solution were collected by centrifugation and resuspended in 1 L of 75% D_2_O M9 medium containing 15 µg mL^−1^ kanamycin. Then, the cells were cultured at 37 °C until OD_600_ = 0.6. The expression of recombinant ER-60 domain fragment was induced by 0.1 m*M* iso­propyl-β-d-thio­galactoside at 16 °C for 64 h. *E. coli* cells were collected by centrifugation at 6500*g* for 20 min at 4 °C. The purification procedure was identical to that used for the hydrogenated domain fragments.

### 2.3. Preparation and activation of *oldenlandia affinis* asparaginyl endopeptidase, *Oa*AEP (C247A)

The recombinant *Oa*AEP (C247A) was expressed using an *E. coli* expression system. Detailed construct information is provided by Okuda *et al.* (2023 ▸). The expression of recombinant *Oa*AEP (C247A) was induced in LB broth containing 0.1 m*M* iso­propyl-β-d-thio­galactoside, 15 µg mL^−1^ kanamycin and 12.5 µg mL^−1^ tetracycline at 16 °C for 64 h. *E. coli* cells were collected by centrifugation at 6500*g* for 20 min at 4 °C, sonicated in 20 m*M* sodium phosphate-buffered saline (pH 8.0) containing 500 m*M* NaCl and centrifuged at 26000*g* for 20 min at 4 °C. The supernatant was applied to a packed column with Ni Sepharose 6 Fast Flow resin (GE Healthcare). The column was washed with 50 m*M* sodium phosphate-buffered saline (pH 8.0) containing 500 m*M* NaCl and 20 m*M* imidazole. The proteins were eluted with 20 m*M* sodium phosphate-buffered saline (pH 8.0) containing 500 m*M* NaCl and 500 m*M* imidazole. To cleave the His_6_-Smt3 tag, the eluted proteins were treated with SUMO protease (LifeSensors) at 4 °C overnight. The cleaved tag was removed from the sample by applying it to His60 Ni Superflow Resin (TaKaRa BIO Inc.). The proteins were subsequently purified by ion-exchange column chromatography on a Resource Q column with 20 m*M* Tris–HCl buffer (pH 8.0). Low-pH exposure is required to activate the ligation activity of *Oa*AEP (C247A) (Yang *et al.*, 2017 ▸). To activate, the purified *Oa*AEP (C247A) (0.5 mg mL^−1^) was dialyzed against 100 m*M* sodium citrate (pH 4.0) containing 150 m*M* NaCl and 1 m*M* EDTA overnight at 4 °C. Insoluble proteins generated during dialysis were removed by centrifugation at 6500*g* for 15 min at 4 °C. The supernatant was purified by Superdex 75 Increase 10/300 GL (GE Healthcare) with 100 m*M* sodium citrate (pH 4.0) containing 150 m*M* NaCl.

### 2.4. Ligation reaction of ER-60 domain fragments with *Oa*AEP (C247A)

For the first-step ligation reaction, ER-60 **bb**′ and **a**′ domain fragments (each at 10 µ*M*) were incubated with 0.2 µ*M* activated *Oa*AEP (C247A) in a reaction buffer consisting of 200 m*M* Tris–HCl (pH 8.0) and 150 m*M* NaCl for 64 h at 20 °C. The reaction time and temperature were selected on the basis of previously optimized conditions (Okuda *et al.*, 2023 ▸). After incubation, the reaction mixture was diluted with 2× Laemmli SDS sample buffer (Laemmli, 1970 ▸) and heated at 95 °C for 5 min. Samples were analyzed by sodium dodecyl sulfate–polyacrylamide gel electrophoresis (SDS-PAGE), and gels were stained with Coomassie Brilliant Blue R-250. Band intensities were quantified using *ImageJ* (National Institutes of Health, USA). Ligation efficiency was calculated as follows:
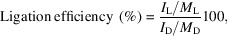
where *I*_L_, *M*_L_, *I*_D_ and *M*_D_ represent the band intensity and molecular weight of the ligation product and the band intensity and molecular weight of the domain fragment in higher quantity within the reaction mixture without *Oa*AEP, respectively.

Because the ligation mixture contained unreacted ER-60 fragments, ligation products and enzyme, the products were purified before further use. Ligation products from the first-step reaction were purified by ion-exchange chromatography on a Resource Q column equilibrated with 20 m*M* Tris–HCl (pH 8.0). Prior to the second-step ligation, the His_6_-Smt3 tag was removed from the **a** domain fragment using SUMO protease followed by His60 Ni Superflow resin (TaKaRa Bio Inc.). For the second-step ligation, ER-60 **a** domain and **bb**′-**a**′ ligation products (10 µ*M* each) were incubated with 0.2 µ*M* activated *Oa*AEP (C247A) under the same conditions as described above. The resulting ligation products were again purified by Resource Q ion-exchange chromatography (20 m*M* Tris–HCl, pH 8.0). For final purification of full-length ligated ER-60, the His_6_-Smt3 tag was removed using SUMO protease and His60 Ni Superflow resin, and the product was subsequently subjected to size-exclusion chromatography on a Superdex 200 Increase 10/300 GL column equilibrated with 20 m*M* Tris–HCl (pH 8.0) containing 150 m*M* NaCl.

Hereafter, the hydrogenated **a** and **a**′ domains are denoted as *h*(**a**) and *h*(**a**′), respectively, and the partially deuterated **bb**′ domain as *pd*(**bb**′). Domains connected by ligation are indicated by hyphens (-). Accordingly, the fully hydrogenated wild-type ER-60 is referred to as *h*(WT), and the hydrogenated ligation-mutant is denoted as *h*(**a**-**bb**′-**a**′).

### 2.5. Matrix-assisted laser desorption/ionization–time of flight mass spectrometry (MALDI-TOF MS)

ER-60 domain samples were mixed with a saturated sinapinic acid matrix solution (Bruker Daltonics, Germany) prepared in TA30 (30% aceto­nitrile in 0.1% TFA aqueous solution) at a sample-to-matrix ratio of 1:9. Aliquots were spotted onto a ground steel MALDI target plate (Bruker Daltonics) and allowed to air-dry and crystallize. Mass spectra were acquired on a microflex LT MALDI-TOF mass spectrometer (Bruker Daltonics) operated in positive-ion mode. External calibration was performed using Protein Standards II (Bruker Daltonics). Spectra were recorded using flexControl and analyzed with the *FlexAnalysis* software (Bruker Daltonics).

### 2.6. SAXS measurement

SAXS measurements were performed using a NANOPIX (Rigaku, Tokyo, Japan). X-rays from a high-brilliance point-focused X-ray generator (MicroMAX-007HF, Rigaku, Tokyo, Japan) were focused with a confocal mirror (OptiSAXS) and collimated with the lower parasitic scattering slit system, ‘ClearPinhole’. The scattered X-rays were detected with a two-dimensional semiconductor detector (HyPix-6000, Rigaku, Tokyo, Japan) with a spatial resolution of 100 µm. Two sample-to-detector distance conditions, 1330 and 300 mm, were used to cover a *q* range of 0.01–0.70 Å^−1^, where *q* = (4π/λ)sin(θ/2) with λ (= 1.54 Å) and θ representing the X-ray wavelength and scattering angle, respectively. The *h*(WT) sample was prepared at a concentration of 4 mg mL^−1^, whereas the *h*(**a**-**bb**′-**a**′) and *h*(**a**)-*pd*(**bb**′)-*h*(**a**′) samples were prepared at 1.5 mg mL^−1^ in 20 m*M* Tris–HCl buffer (pH 8.0) containing 150 m*M* NaCl and 1 m*M* CaCl_2_. Scattering profiles were recorded as 300 s frames, and 72 and 120 frames were merged for low-*q* (0.01–0.06 Å^−1^) and high-*q* (0.06–0.70 Å^−1^) setups, respectively. The obtained two-dimensional scattering patterns were converted to one-dimensional scattering profiles by radial averaging. Subsequently, one-dimensional scattering profiles were corrected for the intensity of the incident beam and the transmission. Then, the scattering profile of the protein in solution was obtained by subtracting that of the buffer. Finally, the scattered intensity was converted to an absolute scale by comparison with the scattering intensity of water (*I*_water_ = 1.632 × 10^−2^ cm^−1^). All data reductions were processed with *SAngler* (Shimizu *et al.*, 2016 ▸).

### 2.7. Analytical ultracentrifugation (AUC)

The AUC experiments were performed at 60000 rpm at 25 °C with a sedimentation velocity method using Rayleigh interference optics (ProteomeLab XL-I, Beckman Coulter). The weight concentration distribution *c*(*s*_20,w_) as a function of the sedimentation coefficient was obtained by fitting the time evolution of sedimentation data with the Lamm formula (Schuck, 2000 ▸) using the *SEDFIT* software (Schuck, 2004 ▸). The sedimentation coefficient was also normalized to the value at 20 °C in pure water (*s*_20,w_).

### 2.8. SEC-SANS measurements

SEC-SANS measurements were performed using the D22 instrument at the Institut Laue–Langevin (Grenoble, France) (Jordan *et al.*, 2016 ▸; Martel *et al.*, 2023 ▸) and the Bio-SANS instrument located at the High Flux Isotope Reactor at Oak Ridge National Laboratory (Oak Ridge, TN, USA) (Heller *et al.*, 2014 ▸; Johansen *et al.*, 2018 ▸; Thomas *et al.*, 2024 ▸).

SEC-SANS measurements on D22 were performed using neutrons with a wavelength of λ = 6 Å and a wavelength spread of Δλ/λ ≃ 10%. By setting the sample-to-detector distance to 8 m for the first detector and 1.4 m for the second detector, the *q* range covered from 0.01 to 0.7 Å^−^^1^. For SEC-SANS measurements at the Bio-SANS instrument, the main detector array was at 7.0 m from the sample, the curved wing detector was fixed at 1.13 m from the sample and rotated to 7.25° from the direct beam, and the curved mid-range detector was fixed at 4 m from the sample and rotated to 2.7° from the direct beam. Using this three-detector array configuration, the *q* ranges obtained in a single exposure using 6 Å neutrons were 0.007 < *q* < 0.9 Å^−1^. The wavelength spread (Δλ/λ) was 13.2%. The source and sample aperture radii were 20 and 7 mm, respectively, and the apertures were separated by 9.7 m. A semitransparent beam trap of borated aluminium of 38 mm radius was used to acquire transmission and scattering data simultaneously.

In both SEC-SANS measurements, a 500 µL volume of sample at 5 mg mL^−1^ was injected onto a Superdex 200 Increase 10/300 GL column (Cytiva). Prior to SEC-SANS measurements, AUC analysis confirmed that approximately 90% of the sample population was monomeric. The running buffer consisted of 20 m*M* Tris–HCl (pH 8.0), 150 m*M* NaCl and 1 m*M* CaCl_2_ in 100% D_2_O. Eluted sample from the SEC column was directly flowed into a flow cell installed on the sample stage of each instrument.

For SEC-SANS measurements on D22, a UV absorbance detector (280 nm) was aligned at 45° relative to the flow-cell window, enabling real-time monitoring of the elution profile at the cell position. This configuration allowed selective data acquisition at the elution peak of the target fraction. Upon detection of the peak at the cell position, the flow was stopped to retain the eluted peak fraction within the cell for extended acquisition. Data were collected until sufficient counting statistics were achieved. Scattering profiles were recorded in 61.5 min (= 123 frames × 0.5 min per frame) and 127.5 min (= 255 frames × 0.5 min per frame) for *h*(WT) and *h*(**a**)-*pd*(**bb**′)-*h*(**a**′), respectively. Under these measurement conditions, the relative error of the radius of gyration (*R*_g_) fell below 0.5% for *h*(WT) and below 1.5% for *h*(**a**)-*pd*(**bb**′)-*h*(**a**′), respectively.

Compared with conventional SEC-SANS measurements under continuous flow, this approach allows longer acquisition times and thereby improves signal-to-noise ratio. The integrated scattering intensity was monitored continuously during measurement to identify the onset of aggregation or degradation. Upon detection of these effects, only data collected before their onset were used to determine the scattering profile of the target fraction.

For Bio-SANS measurements, no UV absorbance detector was available at the flow-cell position. Instead, the UV detector (280 nm) of the high-performance liquid chromatography system (ÄKTA pure) located downstream of the SEC column was used to monitor the elution profile. Because sample concentration could not be monitored at the flow cell, the stop-flow strategy used on D22 was not applicable. Therefore, the flow rate was reduced to 0.02 mL min^−^^1^ during peak elution to increase the residence time within the cell, thereby increasing the residence time of the sample within the beam and enabling longer effective acquisition times. Scattering profiles were recorded in 155 min (= 31 frames × 5 min per frame) for *pd*(**bb**′).

The observed SANS intensity was corrected for background, empty cell and buffer scattering, and transmission, and subsequently converted to the absolute scale using the *GRASP* software (Dewhurst, 2023 ▸) or *drtsans* (Heller *et al.*, 2022 ▸). Data frames collected immediately after the SEC peak top, where stable *I*(*q*) profiles without detectable time-dependent changes were observed, were selected and averaged for analysis. Buffer scattering was subtracted to obtain the scattering profile of the sample.

### 2.9. MD simulations

Molecular models of the initial structures for MD simulations were prepared using *Molecular Operating Environment* (*MOE*) version 2024.0601 (Chemical Computing Group, 2025 ▸). The crystal structure of the tapasin–ER-60 complex (PDB ID 3f8u; Dong *et al.*, 2009 ▸) was used as the basis for extraction of the tapasin and crystal water molecules. Hydrogen atoms were added under pH = 7.0 conditions using the ‘protonate 3D’ function of *MOE*. Energy minimizations were performed with positional constraints applied to the heavy atoms of the modeled residues. Both oxidized and reduced forms between cysteine residues were prepared for ER-60 structures.

All MD simulations were performed using *GROMACS 2021.5* (Abraham *et al.*, 2015 ▸). Topology files were generated with *AmberTools* (Case *et al.*, 2023 ▸). The AMBER ff14SB force field (Maier *et al.*, 2015 ▸) was used for ER-60. Each system was neutralized with Na^+^ counterions and solvated in a periodic box using the TIP3P water model. After energy minimization, the system was gradually heated from 100 to 310.15 K over 1 ns under the NVT ensemble, and then an additional 1 ns simulation was performed at 310.15 K. During this process, positional restraints with a force constant of 1255 kJ mol^−1^ nm^−2^ were applied to the backbone heavy atoms. Subsequently, density relaxation was performed under the NPT ensemble (1.0 bar) for 4 ns while gradually releasing positional constraints as follows: force constants of 1255, 837 and 418 kJ mol^−1^ nm^−2^ were applied to the heavy atoms of the main chain every 1 ns, with no positional constraints applied during the final 1 ns. Finally, ten runs of equilibration for 5 and 100 ns production runs were conducted under the NPT ensemble. These MD simulations were run ten times. Throughout all simulation steps, no constraints were applied to bond lengths. The time step was 0.5 fs for all simulations.

## 3. Results and discussion

### 3.1. Preparation of segmentally deuterated ER-60

**Figure 1:**
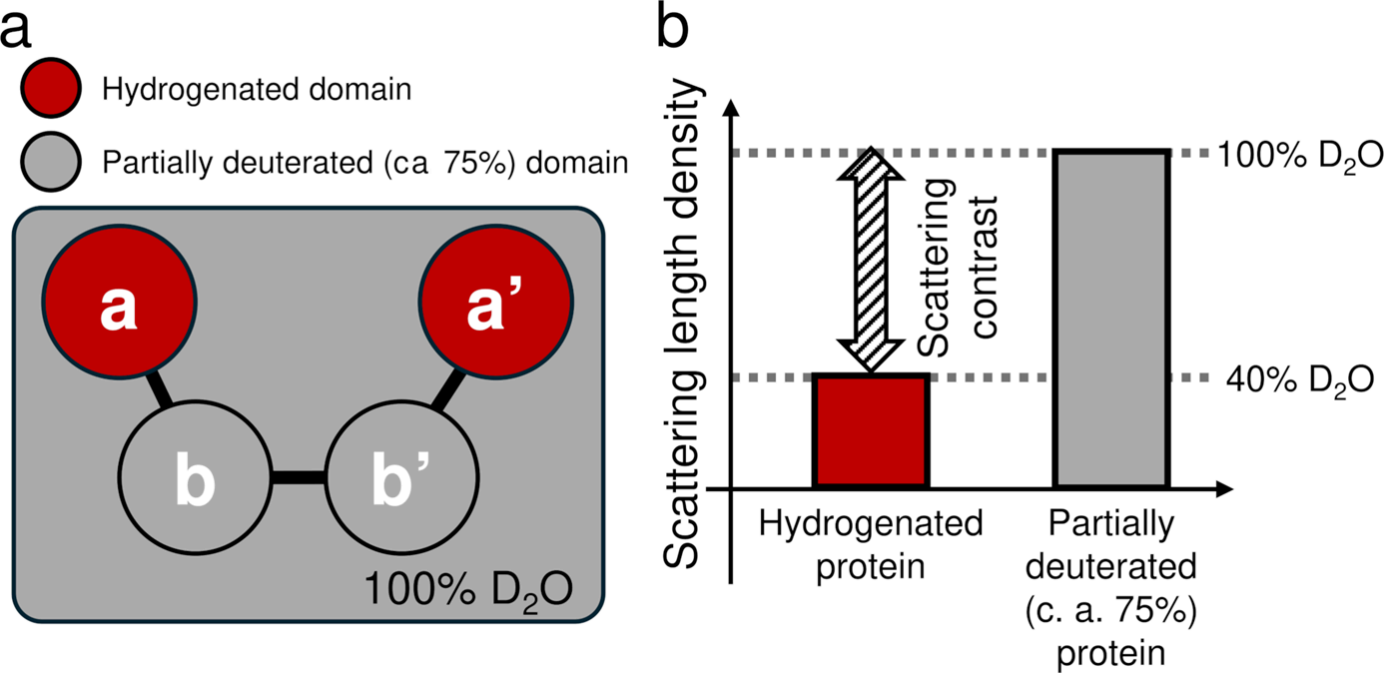
(*a*) Schematic image of segmentally deuterated ER-60 comprising partially deuterated (*ca* 75%) **bb**′ domains (gray) and hydrogenated **a** and **a**′ domains (red). The SLD of partially deuterated **bb**′ domains matches that of 100% D_2_O, rendering them ‘scattering invisible’ in SANS. (*b*) Schematic illustration of SLD of hydrogenated and partially deuterated proteins. The double-headed arrow indicates the difference of SLD between 100% D_2_O and hydrogenated protein, termed the scattering contrast.

As illustrated in Fig. 1 ▸(*a*), ER-60 consists of four thio­redoxin-like domains arranged sequentially in an **a**–**b**–**b**′–**a**′ architecture. Our previous studies have suggested that changes in the relative arrangement of the **a** and **a**′ domains could be coupled to its functional regulation (Okuda *et al.*, 2021*b* ▸). In the present study, we prepared a segmentally deuterated ER-60 in which the spatially separated, non-contiguous **a** and **a**′ domains were hydrogenated, whereas the **b** and **b**′ domains were partially deuterated. In SANS, the scattering contrast, defined as the difference in scattering length density between solute and solvent, governs the scattering intensity. In 100% D_2_O, hydrogenated proteins retain a non-zero scattering contrast, whereas partially deuterated proteins become effectively contrast matched to the solvent [Fig. 1 ▸(*b*)]. Accordingly, in the present ER-60 construct [Fig. 1 ▸(*a*)], the **a** and **a**′ domains are ‘scatteringly visible’, whereas the **bb**′ domains are ‘scatteringly invisible’ in iCM-SANS measurements.

**Figure 2:**
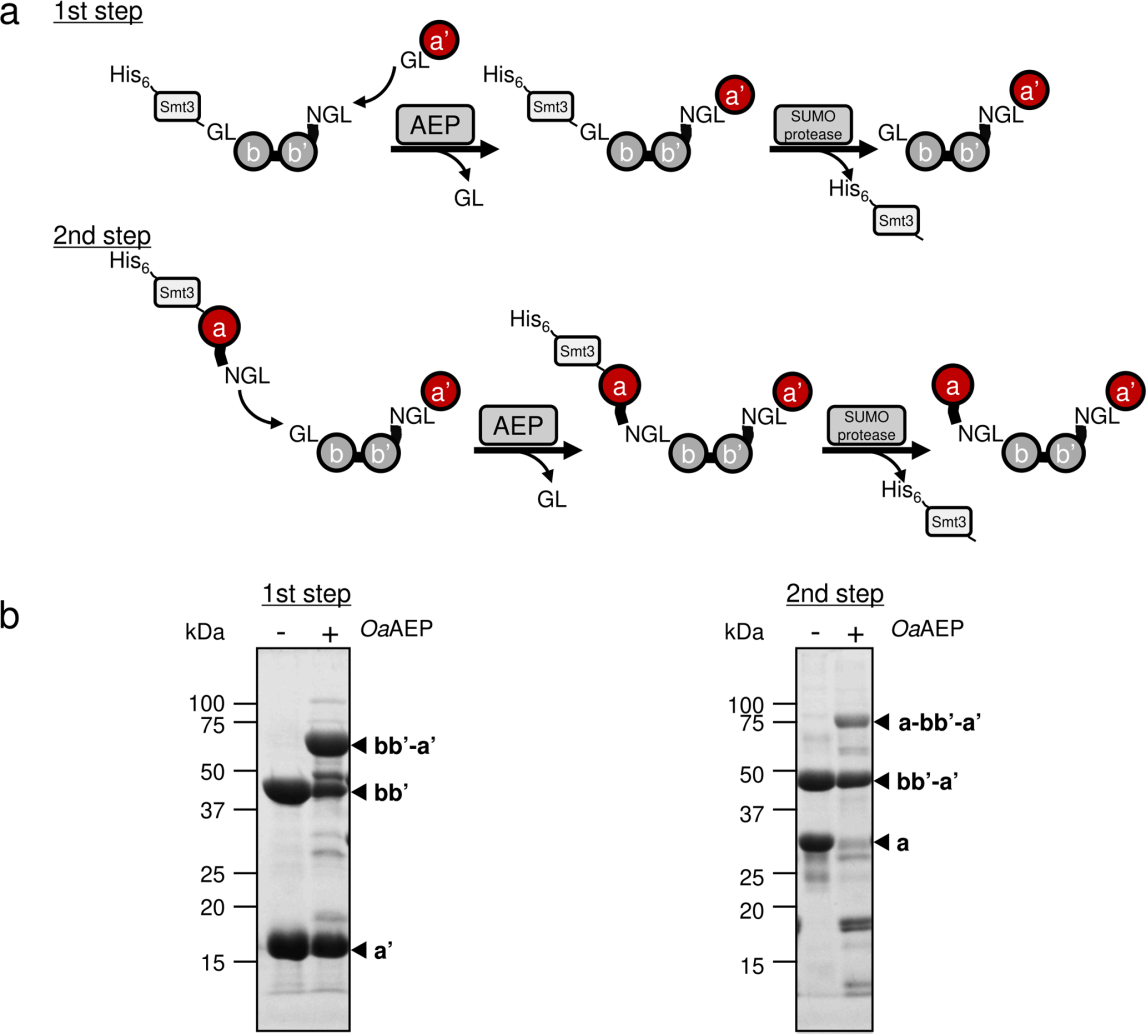
Schematic representation of preparation of segmentally deuterated ER-60 and SDS-PAGE analysis of the first- and second-step ligation reactions. (*a*) In the first step, the **bb**′ and **a**′ domains were ligated using *Oa*AEP to generate **bb**′-**a**′. In the second step, the **a** domain was ligated to **bb**′-**a**′ to reconstruct full-length ER-60. (*b*) SDS-PAGE of the two-step ligation reactions. First step: ligation of **bb**′ and **a**′ domains. Second step: ligation of the **a** domain to **bb**′-**a**′. Lane 1: reaction mixture without *Oa*AEP. Lane 2: reaction mixture with *Oa*AEP.

Segmentally deuterated ER-60 was prepared by separately expressing and purifying the domains of interest under different isotopic conditions. The **a** and **a**′ domains, designated for selective observation with iCM-SANS, were expressed and purified using an *E. coli* expression system under hydrogenated conditions, whereas the **bb**′ domain was expressed and purified under partially deuterated conditions. These individually prepared domains were subsequently reconstituted into a full-length ER-60 through protein ligation using *Oa*AEP (Fig. 2 ▸).

We successfully obtained selectively domain-deuterated ER-60 with the native amino acid sequence, except for the minimal *Oa*AEP recognition sequence (NGL) (Fig. S1). The degree of deuteration of the **bb**′ domain was determined by MALDI-TOF MS to be 72.5%. This value is in close agreement with the theoretical value of 73.9%, which is expected to match the SLD of 100% D_2_O (Fig. S2).

The ligation efficiencies for the segmentally deuterated ER-60 are summarized in Table S1. In the first-step ligation between *pd*(**bb**′) and *h*(**a**′), the reaction efficiency reached 62%. This was comparable to that for ligation performed exclusively between hydrogenated domains (Okuda *et al.*, 2023 ▸), indicating that deuteration of the domain had little effect on the ligation performance. In contrast, the second-step ligation between *h*(**a**) and *pd*(**bb**′)-*h*(**a**′) exhibited a reduced efficiency of 19%, less than half of that in the first step. Nevertheless, when 30–50 mg of each domain fragment was prepared, approximately 2.5 mg of the final segmentally deuterated ER-60 product was obtained. Using our optimized *Oa*AEP-mediated ligation protocol, it is possible to prepare the segmentally deuterated MDPs in practical amounts for iCM-SANS experiments. Additionally, this protocol uniquely presents segmentally deuterated ER-60 suitable for selective observation of spatially separated, non-contiguous domains.

### 3.2. Structural characterization of WT, hydrogenated ligation-mutant and segmentally deuterated ER-60 with AUC and SAXS

To evaluate whether the ligation procedure and the associated sequence mutations perturbed the native structure of ER-60, SAXS and AUC measurements were performed. The AUC analysis revealed the presence of a small fraction of aggregated ER-60 in addition to the monomeric component (Fig. S3 and Table S2). Because such aggregates could affect the precise analysis of scattering profiles, their contributions must be appropriately removed from observed SAXS profiles. Although SEC-SAXS is a common approach for this purpose, it requires a relatively large amount of sample (5 mg mL^−1^, 500 µL), which was not feasible given the limited sample availability for SANS measurements. We therefore applied the AUC-SAS method (Morishima *et al.*, 2020 ▸; Morishima *et al.*, 2023 ▸), which allows both SAXS and AUC measurements with as little as 1.5 mg mL^−1^ and 100 µL of sample, enabling removal of aggregate contributions from the observed SAXS profile. This approach allowed us to obtain the scattering profile corresponding to monomeric ER-60.

**Figure 3:**
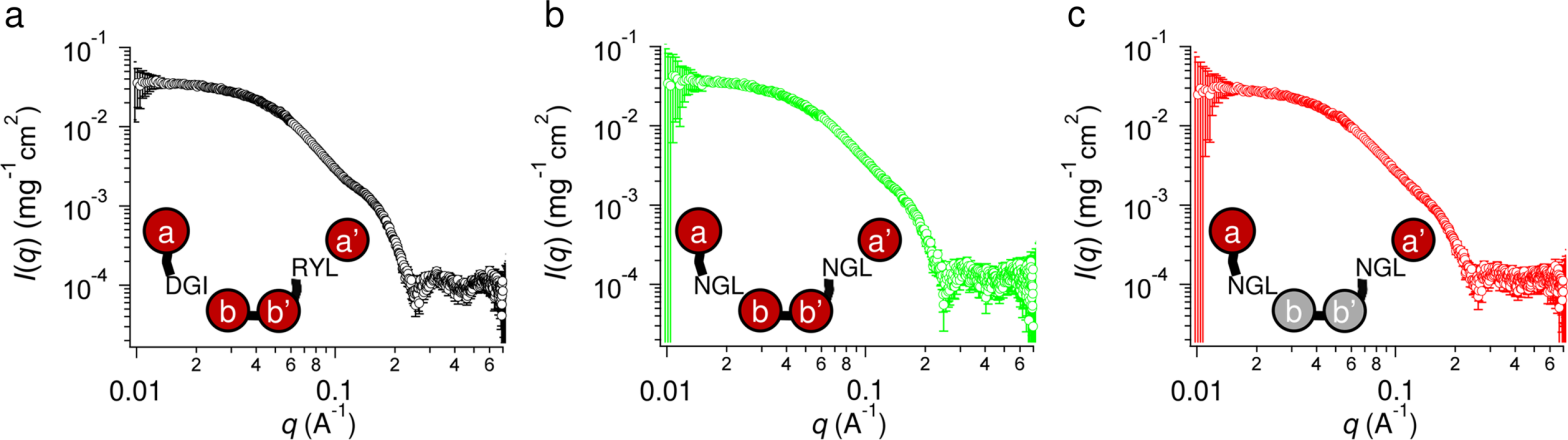
SAXS profiles of ER-60 constructs: (*a*) hydrogenated wild-type ER-60 [*h*(WT)], (*b*) hydrogenated ligation-mutant [*h*(**a**-**bb**′-**a**′)] and (*c*) segmentally deuterated ER-60 [*h*(**a**)-*pd*(**bb**′)-*h*(**a**′)]. Insets show schematic representations of the ER-60 constructs. Red and gray domains indicate hydrogenated and partially deuterated domains, respectively. The SASBDB accession codes for *h*(WT), *h*(**a**-**bb**′-**a**′) and *h*(**a**)-*pd*(**bb**′)-*h*(**a**′) are SASDZT8, SASDZU8 and SASDZS8, respectively.

No significant differences were observed in the overall domain arrangement in solution among wild-type ER-60 [*h*(WT)], the ligation-mutant containing the residual NGL sequence [*h*(**a**-**bb**′-**a**′)] and the segmentally deuterated construct [*h*(**a**)-*pd*(**bb**′)-*h*(**a**′)] (Fig. 3 ▸, Table 1 ▸ and Fig. S4). These results indicate that neither domain ligation nor segmental deuteration induces substantial perturbations to the overall domain arrangement of ER-60. Furthermore, the present segmental deuteration strategy yielded samples of sufficient quantity and quality for SANS measurements even after multi-step ligation. This certifies that domain-selective structural information obtained from iCM-SANS can serve as complementary experimental constraints to the overall structural information from SAXS.

### 3.3. Inverse contrast-matching SEC-SANS (iCM-SEC-SANS) studies on WT and segmentally deuterated ER-60

Since aggregates coexisting in solution can affect detailed analysis of SANS scattering profiles as well, we adopted the SEC-SANS method to remove the contribution of the aggregates from that of sample solution. iCM-SEC-SANS measurements were firstly performed for *pd*(**bb**′) in 100% D_2_O [Figs. S5(*a*) and S5(*b*)]. The results demonstrated that the **bb**′ domains were effectively contrast matched, rendered ‘scatteringly invisible’ in iCM-SEC-SANS measurements. Namely, only the spatially separated, non-contiguous **a** and **a**′ domains are selectively observable from iCM-SEC-SANS.

AUC analysis confirmed the presence of minor non-monomeric species in all samples (Fig. S3 and Table S2). To minimize the contribution of these aggregated species to the SANS profiles, data acquisition was performed such that fractions at elution volumes slightly higher than the peak top, where the contribution of aggregated components is minimal, flowed into the measurement cell. To quantitatively assess the contribution of aggregated species near the peak top, Gaussian peak fitting analysis was applied to the SEC elution profiles, confirming that the contribution of aggregated components was at the baseline level in the fractions used for analysis [Figs. S5(*c*) and S5(*d*) and Table S2]. This confirms that the fractions used for SEC-SANS measurements were not contaminated by aggregates.

**Figure 4:**
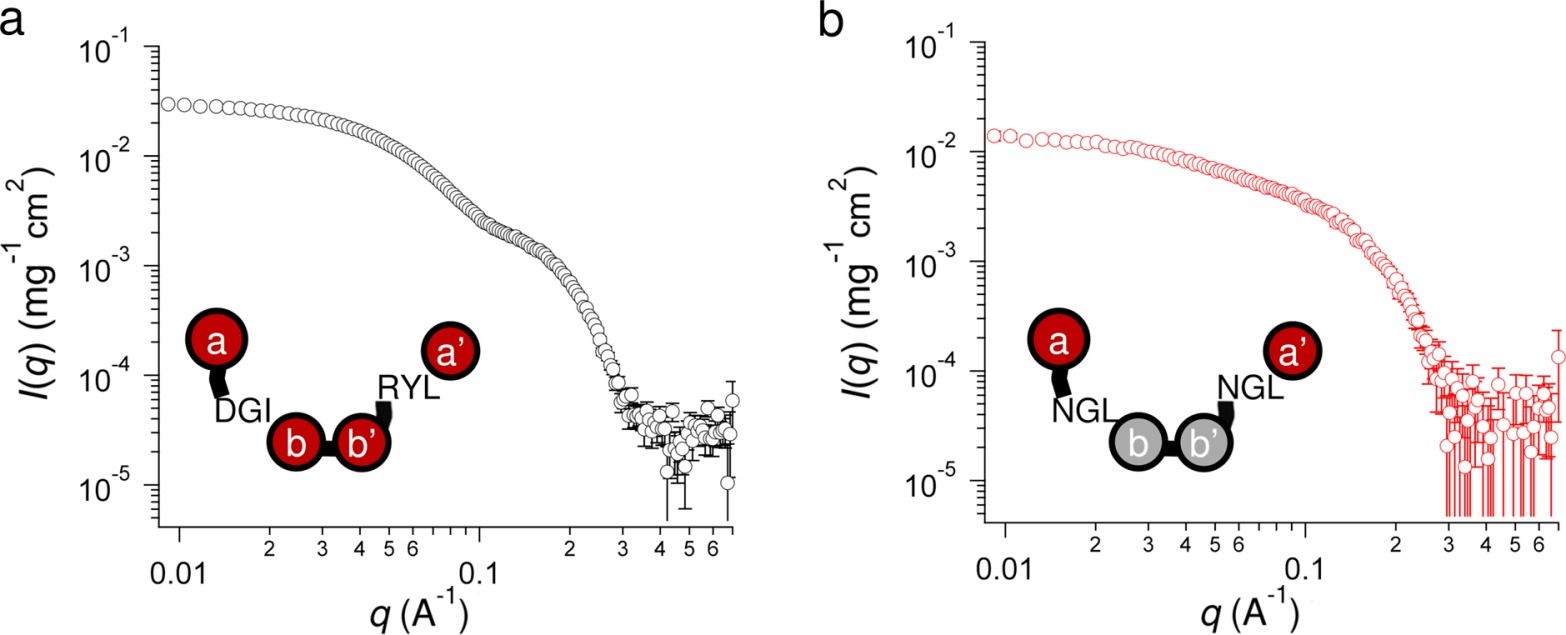
SANS profiles of ER-60 constructs: (*a*) *h*(WT), (*b*) *h*(**a**)-*pd*(**bb**′)-*h*(**a**′). Insets show schematic representations of the ER-60 constructs. Red and gray domains represent hydrogenated and partially deuterated domains, respectively.

We then performed iCM-SEC-SANS measurements for *h*(WT) and *h*(**a**)-*pd*(**bb**′)-*h*(**a**′) in 100% D_2_O (Fig. 4 ▸, Table 2 ▸ and Fig. S6). SEC chromatograms recorded before SANS [Figs S5(*c*) and S5(*d*)], together with scattering intensity and absorbance traces acquired during iCM-SEC-SANS [Figs. S5(*e*) and S5(*f*)], confirmed clear separation of aggregated species from the monomeric target component. These results indicate that scattering contributions from aggregates were effectively removed by SEC. For *h*(WT), the overall structure of ER-60 was observed. In contrast, for *h*(**a**)-*pd*(**bb**′)-*h*(**a**′), the scattering profile reflects the relative arrangement of the **a** and **a**′ domains. No distinct peak corresponding to the center-of-mass distance correlation between the **a** and **a**′ domains was detected. Our previous studies suggested that these domains adopt diverse conformations (Okuda *et al.*, 2021*b* ▸). The absence of a correlation peak in the iCM-SANS profile provides direct experimental evidence that the **a** and **a**′ domains do not adopt a single static or narrowly distributed arrangement in solution, demonstrating that they undergo pronounced conformational fluctuations.

Furthermore, Guinier analysis (Fig. S6) revealed significant differences in *R*_g_ and the forward scattering intensity (*I*_0_) between *h*(WT) and *h*(**a**)-*pd*(**bb**′)-*h*(**a**′) (Table 2 ▸). The marked decrease in *I*_0_ reflects the reduced scattering contrast of *h*(**a**)-*pd*(**bb**′)-*h*(**a**′) relative to the 100% D_2_O buffer. Importantly, the observed *I*_0_ ratio between *h*(WT) and *h*(**a**)-*pd*(**bb**′)-*h*(**a**′) agrees with the calculated value. In contrast, the decrease in *R*_g_ might reflect conformations in which the **a** and **a**′ domains adopt closer proximity.

These results demonstrate that segmental deuteration combined with iCM-SANS enables experimental observation of specific domains in MDPs, thereby providing direct structural information on individual domain arrangements and dynamics.

### 3.4. Discrimination of MD trajectories using complementary experimental constraints

The iCM-SANS measurements of *h*(**a**)-*pd*(**bb**′)-*h*(**a**′) enabled acquisition of domain-specific structural information that cannot be obtained from SAXS alone. However, independent MD simulations can generate distinct conformational ensembles. It was therefore necessary to examine whether such partial structural information provides complementary constraints to SAXS profiles and can effectively contribute to the selection of conformational ensembles derived from MD simulations. To examine this point, we performed ten independent 100 ns MD simulations of ER-60.

**Figure 5:**
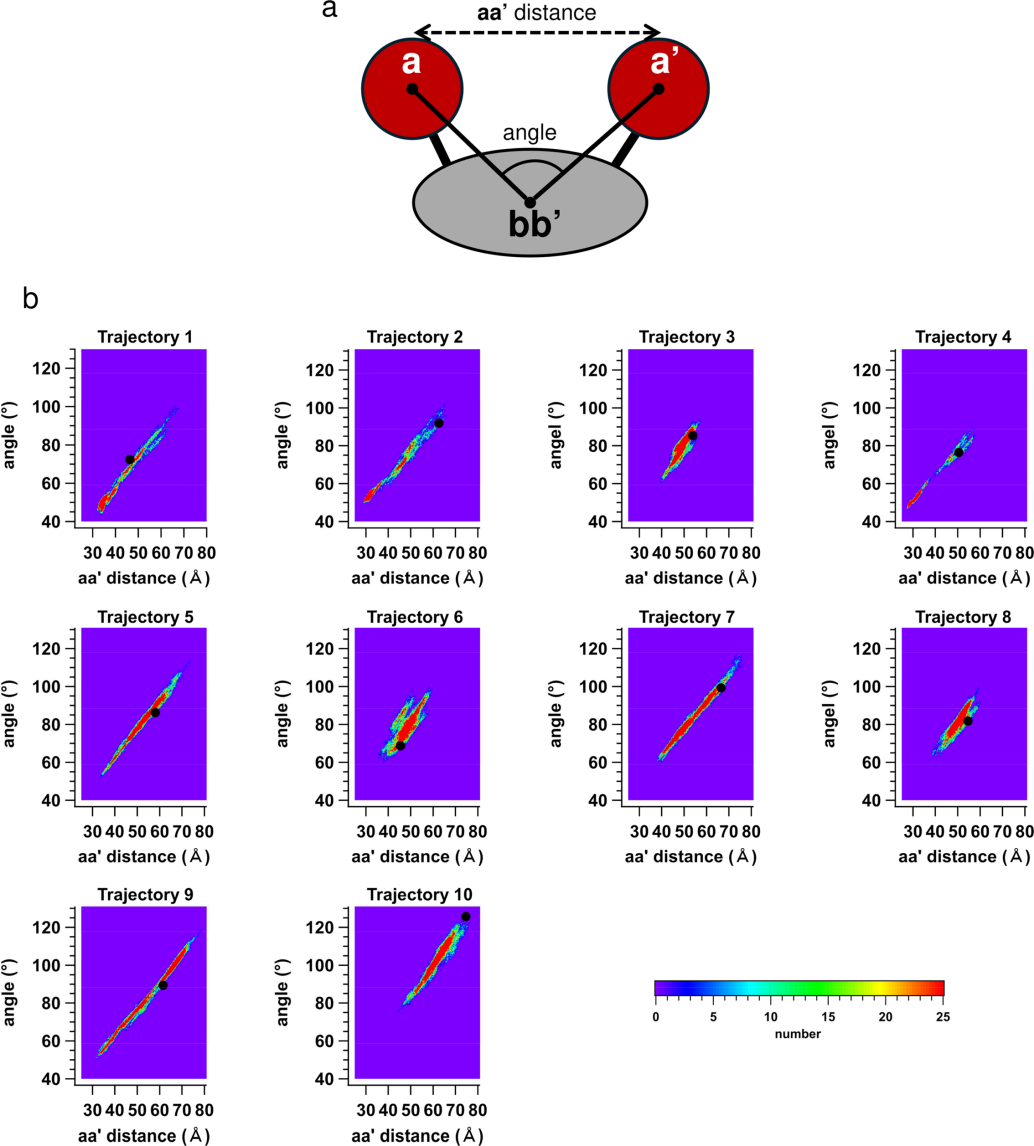
Conformational distributions derived from ten independent MD trajectories. (*a*) Black circles denote domain centers of mass. Distance is defined between the **a** and **a**′ domain centers, and angle as that formed by the **a**–**bb**′–**a**′ centers. These parameters were used to compute conformational distributions for each trajectory. (*b*) 2D distributions of **aa**′ distance and angle for trajectories 1–10; the black circle marks the first data point from the MD production run.

From each trajectory, we calculated (i) the inter-domain distance between the **a** and **a**′ domains and (ii) the angle defined by the center of mass of the **a**, **bb**′ and **a**′ domains (θ**_a_**_-__**bb**′__-__**a**′_). The resulting structural ensembles defined by these two parameters are shown in Fig. 5 ▸(*b*).

**Figure 6:**
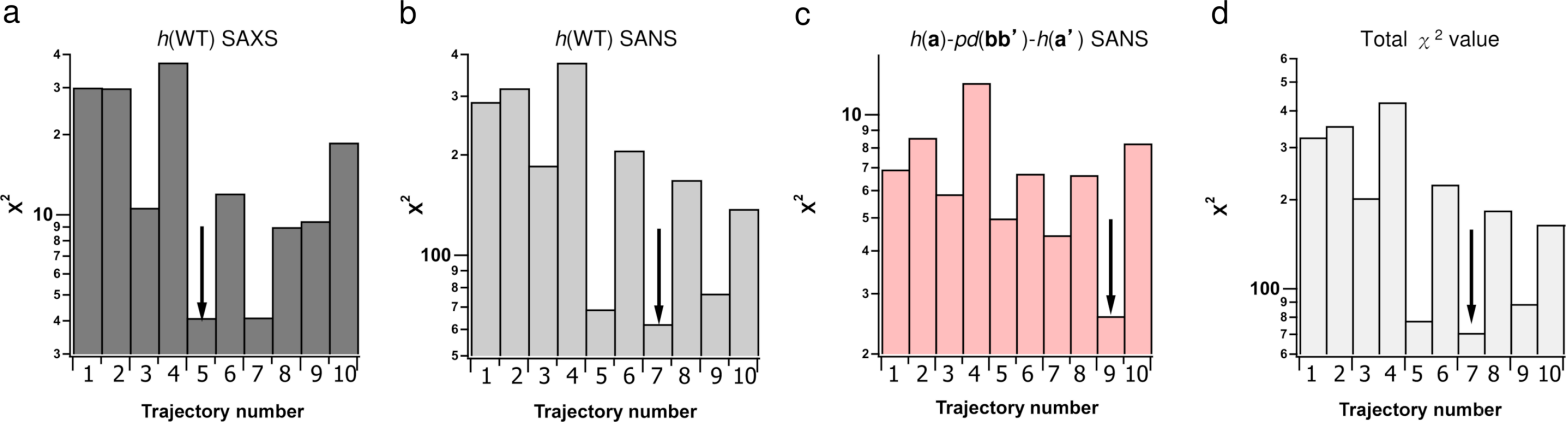
χ^2^ values of independent MD trajectories against SAXS and SANS of *h*(WT) and SANS of *h*(**a**)-*pd*(**bb**′)-*h*(**a**′). Arrows indicate the lowest χ^2^ values, corresponding to the best agreement with experimental profiles. (*a*–*c*) χ^2^ values for individual datasets: agreement between calculated scattering from each trajectory and experimental profiles of (*a*) SAXS of *h*(WT), (*b*) SANS of *h*(WT) and (*c*) SANS of *h*(**a**)-*pd*(**bb**′)-*h*(**a**′). (*d*) Total χ^2^, which is the addition of χ^2^ across all datasets.

**Figure 7:**
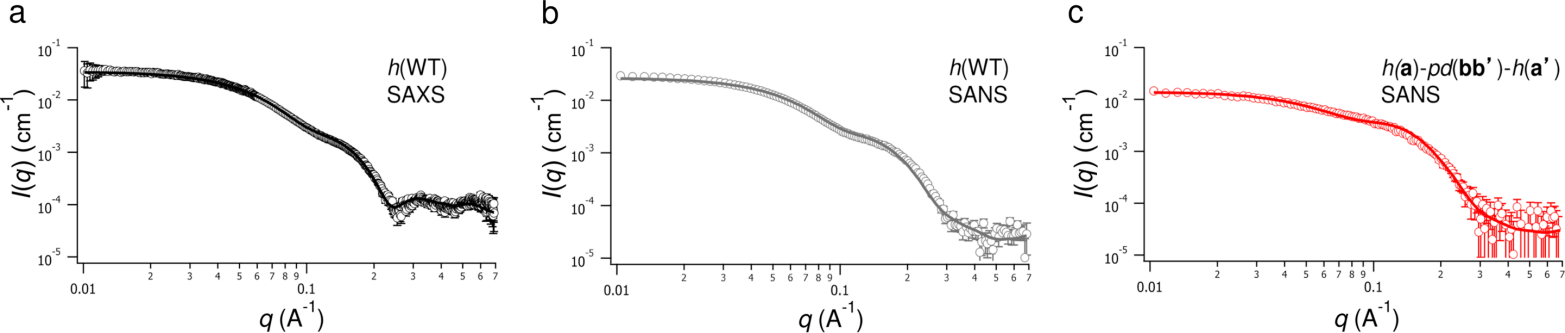
Comparison of experimental scattering profiles with ensemble-averaged theoretical profiles derived from trajectory 7. (*a*) SAXS profile of *h*(WT), (*b*) SANS profile of *h*(WT) and (*c*) SANS profile of *h*(**a**)-*pd*(**bb**′)-*h*(**a**′). Experimental data (open symbols) are overlaid with ensemble-averaged theoretical profiles (solid lines) calculated from trajectory 7.

A total of 100001 snapshots were extracted at 1 ps intervals from each trajectory. Then, theoretical SAXS and SANS profiles of ER-60, as well as the SANS profile of segmentally deuterated ER-60, were calculated using *Pepsi-SAXS* (Grudinin *et al.*, 2017 ▸) or *Pepsi-SANS* ( Grudinin *et al.*, 2021 ▸) from each snapshot. Averaging over all 100001 profiles yielded ensemble-averaged SAXS and SANS profiles. The same procedure was applied separately to each trajectory, resulting in a total of 30 averaged scattering profiles (ten trajectories × three experimental conditions). Using these averaged profiles, χ^2^ values were calculated against the SAXS profile of *h*(WT), the SANS profile of *h*(WT) and the SANS profile of *h*(**a**)-*pd*(**bb**′)-*h*(**a**′) [Figs. 6 ▸(*a*)–6 ▸(*c*)]. As shown in Fig. 6 ▸(*a*)–6 ▸(*c*), the trajectory with the minimum χ^2^ value differed depending on which reference scattering curve was used for comparison: SAXS of *h*(WT), SANS of *h*(WT) or SANS of *h*(**a**)-*pd*(**bb**′)-*h*(**a**′). The best-fit trajectories for SAXS of *h*(WT), SANS of *h*(WT) and SANS of *h*(**a**)-*pd*(**bb**′)-*h*(**a**′) are 5, 7 and 9, respectively. This indicates that selecting the optimal trajectory solely on the basis of a single scattering profile may not be appropriate. We therefore evaluated the total χ^2^, defined as the sum of the χ^2^ values obtained for the three experimental datasets [Fig. 6 ▸(*d*)]. Trajectory 7 yielded the lowest total χ^2^ value and was thus considered the most consistent with the combined experimental constraints within the scope of the present test simulations. A comparison between the ensemble-averaged scattering profiles derived from trajectory 7 and the experimental data is shown in Fig. 7 ▸. Although the experimental curves are qualitatively reproduced, the χ^2^ values indicate that further refinement remains necessary. These results suggest that more extensive MD simulations, including longer sampling times and force-field optimization, are required to adequately capture the real conformational ensemble of highly dynamic MDPs such as ER-60. Nevertheless, the present proof-of-concept analysis demonstrates that incorporation of domain-selective SANS data as complementary constraints alongside SAXS allows effective discrimination among multiple MD trajectories. In other words, the integrated strategy combining SAXS, segmentally deuterated iCM-SANS and MD simulations constitutes a viable protocol for structural ensemble analysis with improved conformational discrimination of MDPs. This protocol is expected to be broadly applicable to other MDPs with more complex architectures.

## 4. Summary

In this study, we developed an integrated protocol for analysis of MDP dynamics by combining segmental deuteration attained by high-efficiency multi-step protein ligation, iCM-SANS, SAXS and MD simulations. Using ER-60 as a model MDP, we successfully prepared segmentally deuterated ER-60 that enables selective observation of spatially separated, non-contiguous domains without perturbing the native structure in solution. Incorporation of domain-selective SANS data alongside SAXS enabled effective discrimination among independent MD trajectories, demonstrating that complementary experimental constraints are essential for resolving degeneracy in ensemble analysis of highly dynamic MDPs. Although extended simulations and further refinement will be required for fully quantitative ensemble determination, the present protocol supports a viable framework for integrating complementary scattering experiments with MD simulations. Importantly, this strategy is broadly applicable to other complex MDPs in which long-range inter-domain dynamics govern function. Looking forward, integration of this approach with high-resolution techniques such as NMR, which provides residue-level dynamical information, and electron paramagnetic resonance, which can provide information on inter-domain distance distributions and conformational heterogeneity through site-directed spin labeling, may further enhance ensemble discrimination and enable a more comprehensive description of domain–domain correlations of MDP in solution. Such multimodal integration represents a promising direction toward quantitative and mechanistic understanding of complex protein dynamics.

## Supplementary Material

Supporting Information. DOI: 10.1107/S1600576726005832/jo5136sup1.pdf

## Figures and Tables

**Table 1:** *R*_g_ and *I*_0_ values derived from SAXS profiles of ER-60 constructs

	*R*_g_ (Å)	*I*_0_ (mg^−1^ cm^2^)
*h*(WT)	32.0 ± 0.2	(3.77 ± 0.38) × 10^−2^
*h*(**a**-**bb**′-**a**′)	32.2 ± 0.4	(3.98 ± 0.40) × 10^−2^
*h*(**a**)-*pd*(**bb**′)-*h*(**a**′)	32.4 ± 0.3	(3.13 ± 0.31) × 10^−2^

**Table 2:** *R*_g_ and *I*_0_ values derived from SANS profiles of ER-60 constructs

	*R*_g_ (Å)	*I*_0_ (mg^−1^cm^2^)
*h*(WT)	32.9 ± 0.1	(2.97 ± 0.30) × 10^−2^
*h*(**a**)-*pd*(**bb**′)-*h*(**a**′)	30.0 ± 0.4	(1.33 ± 0.13) × 10^−2^

## Data Availability

The data that support the findings of this study are available from the corresponding author upon reasonable request. The SAXS datasets for *h*(WT), *h*(**a**-**bb**′-**a**′) and *h*(**a**)-*pd*(**bb**′)-*h*(**a**′) have been deposited in SASBDB under accession numbers SASDZT8, SASDZU8 and SASDZS8 and are publicly available at https://www.sasbdb.org/data/SASDZT8, https://www.sasbdb.org/data/SASDZU8 and https://www.sasbdb.org/data/SASDZS8, respectively.
